# Screening of Human Gut Bacterial Culture Collection Identifies Species That Biotransform Quercetin into Metabolites with Anticancer Properties

**DOI:** 10.3390/ijms22137045

**Published:** 2021-06-30

**Authors:** Ranjini Sankaranarayanan, Prabhjot Kaur Sekhon, Achuthan Ambat, Julia Nelson, Davis Jose, G. Jayarama Bhat, Joy Scaria

**Affiliations:** 1 Department of Pharmaceutical Sciences and Translational Cancer Research Center, College of Pharmacy and Allied Health Professions, South Dakota State University, Brookings, SD 57007, USA; ranjini.sankaranarayanan@sdstate.edu; 2 Department of Veterinary & Biomedical Sciences, South Dakota State University, Brookings, SD 57007, USA; prabhjotkaur.sekhon@sdstate.edu (P.K.S.); achuthan.ambat@sdstate.edu (A.A.); julie.nelson@sdstate.edu (J.N.); 3 Department of Chemistry and Physics, Monmouth University, West Long Branch, NJ 07764, USA; djose@monmouth.edu

**Keywords:** chemoprevention, colorectal cancer, flavonoids, quercetin, gut microbiome, bioactive metabolites, probiotics, gut health

## Abstract

We previously demonstrated that flavonoid metabolites inhibit cancer cell proliferation through both CDK-dependent and -independent mechanisms. The existing evidence suggests that gut microbiota is capable of flavonoid biotransformation to generate bioactive metabolites including 2,4,6-trihydroxybenzoic acid (2,4,6-THBA), 3,4-dihydroxybenzoic acid (3,4-DHBA), 3,4,5-trihyroxybenzoic acid (3,4,5-THBA) and 3,4-dihydroxyphenylacetic acid (DOPAC). In this study, we screened 94 human gut bacterial species for their ability to biotransform flavonoid quercetin into different metabolites. We demonstrated that five of these species were able to degrade quercetin including *Bacillus glycinifermentans*, *Flavonifractor plautii*, *Bacteroides eggerthii*, *Olsenella scatoligenes* and *Eubacterium eligens*. Additional studies showed that *B. glycinifermentans* could generate 2,4,6-THBA and 3,4-DHBA from quercetin while *F. plautii* generates DOPAC. In addition to the differences in the metabolites produced, we also observed that the kinetics of quercetin degradation was different between *B. glycinifermentans* and *F. plautii*, suggesting that the pathways of degradation are likely different between these strains. Similar to the antiproliferative effects of 2,4,6-THBA and 3,4-DHBA demonstrated previously, DOPAC also inhibited colony formation ex vivo in the HCT-116 colon cancer cell line. Consistent with this, the bacterial culture supernatant of *F. plautii* also inhibited colony formation in this cell line. Thus, as *F. plautii* and *B. glycinifermentans* generate metabolites possessing antiproliferative activity, we suggest that these strains have the potential to be developed into probiotics to improve human gut health.

## 1. Introduction

In the past, clinical and epidemiological studies largely focused on the ability of flavonols to prevent cancer. Of these flavonols, quercetin has been extensively studied not only for its anticancer effects, but also for its anti-inflammatory, antithrombotic, anti-neurodegenerative, anti-infectious and immunomodulatory activities [1,2]. Quercetin is one of the most abundant flavonoids in the diet and can be found in many foods including onions, apples, grapes, berries, citrus fruits, tea, cherries and broccoli. It has been estimated that humans consume 10–100 mg of quercetin every day on average through their diet [1]. The reported health benefits are predicted to occur through quercetin’s (a) interaction with cellular receptors, (b) modification of signal transduction pathways, (c) antioxidant properties and (d) apoptosis regulation, to name a few [3,4,5].

Quercetin, however, is not freely present as an aglycone in most of these food sources. It is usually conjugated to a sugar moiety (forming glycosides) that confers water solubility and chemical stability to these compounds [6]. The sugar moiety attached to quercetin is usually glucose or rhamnose, but it could also be galactose, arabinose, xylose or other sugars [7]. Quercetin glycosides are poorly absorbed from the intestines, and hence these linkages are cleaved by enzymes present either in the small intestine or the colon to facilitate absorption [8,9]. As the enzymes produced by the human body are sometimes incapable of cleaving the glycosidic linkages, microbial enzymes produced in the gut have been implicated in these deconjugation reactions. For example, studies have suggested that the hydrolysis of rutin (quercetin-3-O-rutinoside; from onions, berries, etc.) to quercetin occurs through the action of gut microbiota [10,11]. Studies highlighting the importance of gut microflora in human health are now emerging, wherein microbiota is considered as a separate organ in itself [12,13,14,15].

Numerous studies have implicated the dysregulation of the microbial ecology in the intestines to be a cause of colorectal cancers (CRC). These studies have also indicated that diet is a major factor governing dysbiosis wherein regular intake of fruits and vegetables seems to favorably shift the ecology of gut microbes to a cohort that can prevent the occurrence of CRC [16,17,18,19,20,21]. Increasingly, studies are also implicating a beneficial role of microbial metabolites in human health, especially in cancer [22,23,24]. Taken together, these studies are indicative of the importance of gut microbes and their flavonoid catabolites in influencing human health. Hence, identifying the mechanisms involved in flavonoid catabolism and the various microbial enzymes involved in this process would aid in understanding and establishing the key contributors to the generation of beneficial metabolites.

Studies conducted thus far have identified quercetin-2,3-dioxygenase (quercetinase) as the first enzyme in *Bacillus* spp. that is required for the metabolism of quercetin [25]. Cleavage of quercetin by this enzyme followed by the action of gut esterases on the intermediary product (2-(3,4-dihydroxybenzoyloxy)-4,6-dihydroxybenzoate) can yield 2,4,6-trihydroxybenzoic acid (2,4,6-THBA) and 3,4-dihydroxybenzoic acid (3,4-DHBA) which are shown to have antiproliferative properties in cancel cell lines. Other studies have also demonstrated that alternative pathways may also exist for quercetin degradation, generating 3,4-dihydroxyphenylacetic acid (DOPAC) through biotransformation of this flavonoid by other bacteria [23,26]. The generation of DOPAC from quercetin is predicted to occur through the involvement of phloretin hydrolase [27]. A recent study also suggested the involvement of ene-reductases in the biotransformation of flavones and flavonols [28]. Apart from these enzymes, other enzymes known to be involved in flavonoid metabolism include chalcone isomerase and enoate reductase from *Eubacterium ramulus* [29] as well as peroxidases, dehydrogenases, demethylases and tyrosinases from a variety of bacteria [30].

We previously demonstrated the ability of 2,4,6-THBA, one of the metabolites generated by flavonoid degradation by gut microbiota, to inhibit cancer cell growth, possibly through inhibition of CDK enzymes [24]. This provided a basis for the hypothesis that human gut microbiota may be crucial for the generation of anticancer metabolites from flavonoids. The goal of this study was to perform microbiome-wide screening to identify the bacteria capable of biotransforming quercetin, a flavonol, and identify the metabolites generated with a focus on 2,4,6-THBA, 3,4-DHBA and DOPAC. This study also focused on the kinetics of quercetin degradation by these bacterial strains and the effect of the generated metabolites on cancer cell proliferation. The findings from this study suggest that gut microbiota may have a role to play in the chemopreventive properties of flavonoids.

## 2. Results

### 2.1. Screening the Human Gut Bacterial Library for Quercetin-Degrading Strains

**Figure 1:**
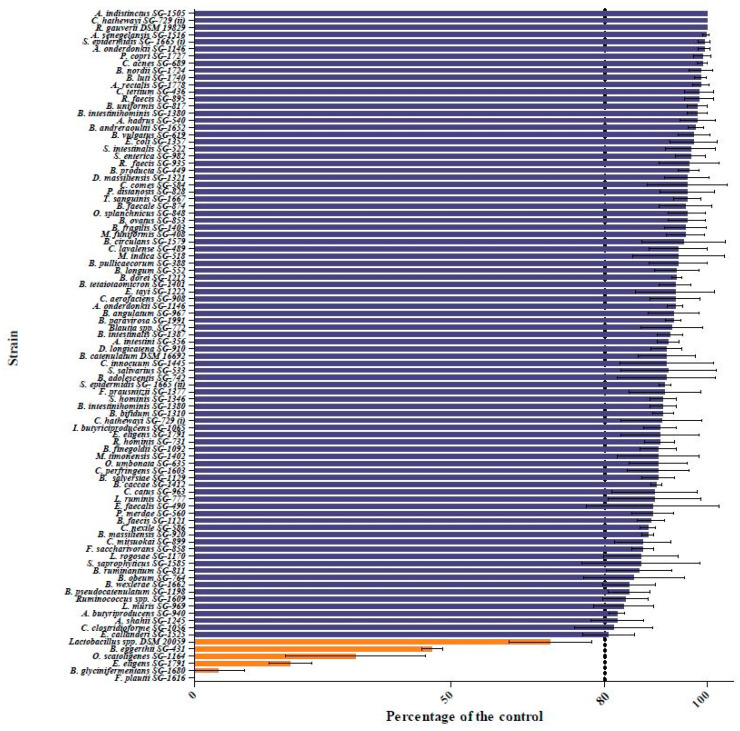
Quercetin degradation by the human gut bacterial library. The figure shows the quercetin-biotransforming capability of each bacterial species as the percentage of degradation when compared to the control. The results indicate that five species (in orange) of the 94 strains tested exhibited >20% quercetin degradation. *Lactobacillus* spp. DSM 20059 served as the positive control.

To identify the species capable of quercetin biotransformation, we screened 91 bacterial species from the human gut microbiota culture library developed by our group [31]. One species known to biotransform quercetin along with two other species not involved in quercetin biotransformation was obtained from DSMZ, Germany, and served as the positive and negative controls, respectively. All the species were individually screened for their ability to degrade quercetin under anaerobic conditions in a 7N minimal medium containing 75 μg/mL quercetin (7NQ medium) as described in the Materials and Methods. To ensure that the quercetin peak did not coincide with any other components present in the spent medium, we also grew the cells in the 7N medium and analyzed the spent supernatant through HPLC. We also included a blank 7NQ medium (without any bacterial inoculation; control) to account for autodegradation of quercetin. The results obtained from the bacterial spent medium were compared to that of the control 7NQ medium, and the results are represented as the percentage of the control. As can be observed in Figure 1, five species presented the ability to degrade >20% of the quercetin supplemented to the medium (in orange) under the experimental conditions. These species were *Bacillus glycinifermentans*, *Flavonifractor plautii*, *Bacteroides eggerthii*, *Olsenella scatoligenes* and *Eubacterium eligens*. The chromatograms for HPLC to detect quercetin degradation for the five bacterial species obtained from the human gut library along with the control are shown in Figure S1.

**Figure 2:**
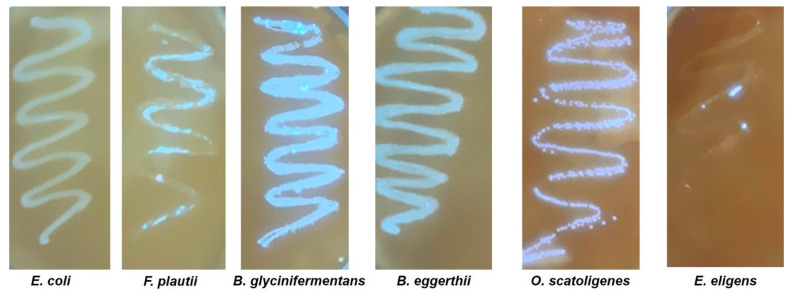
DPH assay demonstrating the ability of five bacterial species to biotransform quercetin. The figure shows bacterial growth on a nylon membrane soaked in a mixture of 1 mM DPH and 20 mM quercetin in mBHI agar plates. All the five quercetin-biotransforming bacterial species presented fluorescence, although to different degrees. However, the negative control, *E. coli* (non-quercetin-degrading bacteria), did not result in any fluorescence in this assay.

To further confirm the quercetin biotransformation ability of the five species, a 1,6-diphenyl-1,3,5-hexatriene (DPH) assay was performed [32]. This assay utilizes the fluorescent compound DPH which can be quenched by quercetin. Degradation of quercetin by the bacteria results in the reversal of this quenching, and quercetin-biotransforming species appear fluorescent under UV light. As shown in Figure 2, fluorescence was observed for all the five species. The negative control, *E. coli*, did not exhibit any fluorescence in these assays. However, for the positive species, the degree of florescence observed in the DPH assay did not completely correspond to the amount of quercetin degradation determined using HPLC. This is possibly because the DPH assay was performed using BHI agar plates while HPLC screening was performed in the 7NQ medium.

### 2.2. HPLC Analysis of Bacillus Glycinifermentans and Flavonifractor Plautii Revealed Different Metabolites Produced Following Quercetin Degradation

**Figure 3:**
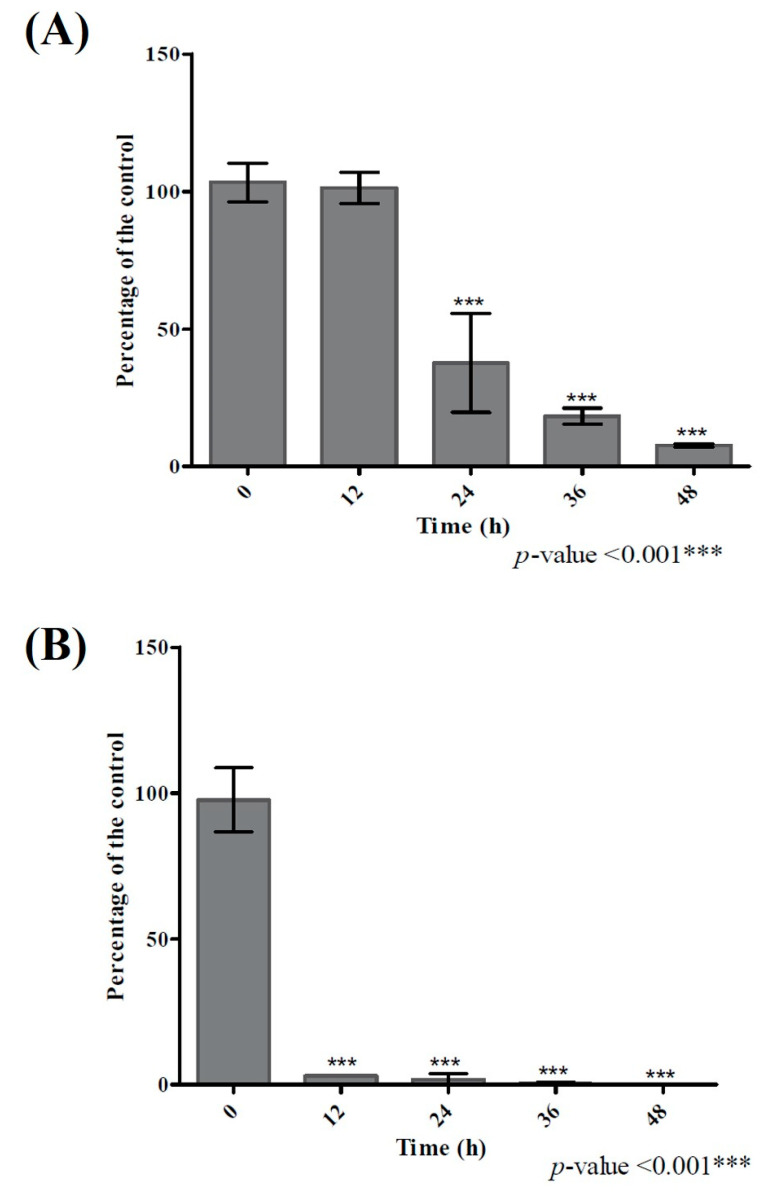
Quercetin degradation patterns of different bacterial species. Kinetics of quercetin degradation by (**A**) *B. glycinifermentans* and (**B**) *F. plautii*.

Among the five bacterial species that could biotransform quercetin (>20%), *Bacillus glycinifermentans* (*B. glycinifermentans*) and *Flavonifractor plautii* (*F. plautii*) exhibited the maximum quercetin degradation under anaerobic incubation for 48 h (Figure 1). Therefore, we decided to focus on these two strains for their ability to biotransform quercetin under the experimental conditions. It is important to note that while *B. glycinifermentans* is a newly reported bacterium [33,34] present in the human gut, its abundance in the human gut is not yet fully characterized. On the other hand, *F. plautii* appears to be fairly distributed in individuals as reported in the literature [35]. It is also important to highlight that under the culture conditions, the growth of *B. glycinifermentans* was profuse while that of *F. plautii* was sparse (Figure S2). As demonstrated in Figure S2, peak growth occurred at 21 h and subsequently decreased after 36 h for *B. glycinifermentans*. On the other hand, the growth pattern of *F. plautii* was slower but sustained over the time period examined. Despite the poor growth that *F. plautii* exhibited, it was able to degrade quercetin much faster than *B. glycinifermentans*. The time course of the pattern of degradation of quercetin by these two bacterial strains is shown in Figure 3. As can be seen, degradation of the supplemented quercetin was almost complete at 48 h for *B. glycinifermentans*, while this was achieved within 12 h by *F. plautii*.

**Figure 4:**
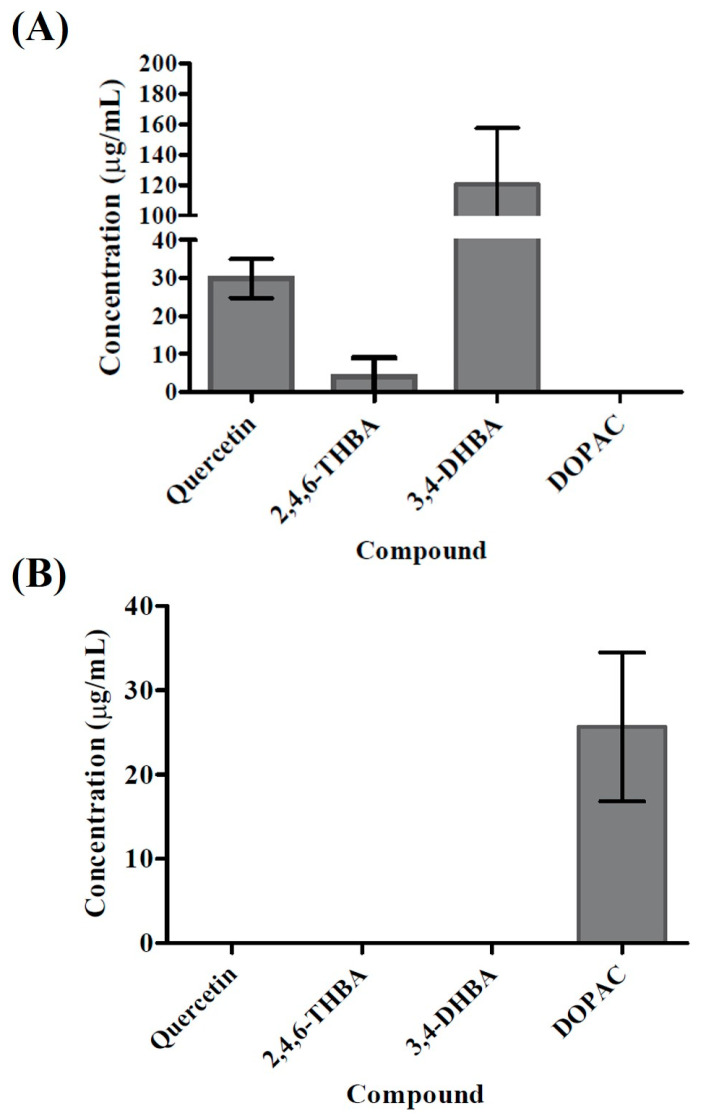
Detection of the metabolites generated by *B. glycinifermentans* (**A**) and *F. plautii* (**B**) following quercetin degradation. HPLC revealed that *B. glycinifermentans* generated 2,4,6-THBA and 3,4-DHBA (**A**) while *F. plautii* generated DOPAC (**B**) upon exposure to quercetin for 24 h. The concentration of each product estimated through standard curves using pure compounds is expressed in μg/mL.

We used UV-visible spectroscopy of the spent medium to identify the metabolites resulting from biotransformation of quercetin by these two species (Figure S3). Absence of the peak at 377 nm in the metabolites of both species confirmed degradation of quercetin by both species. Further analysis of the data with the standards revealed that the metabolites from both species presented a peak around 260 nm which might have come from either 3,4-DHBA or 2,4,6-THBA. In addition, the possibility of DOPAC in these metabolites was detected as the spectra from the metabolites presented a broad peak ranging from 240 nm to 325 nm. A detailed biophysical analysis is currently underway to identify the metabolites observed under different conditions. Further analysis of the spent medium for both species using HPLC revealed that each species produced different metabolites upon incubation with quercetin. 2,4,6-THBA and 3,4-DHBA were clearly detectable in the spent medium isolated from the *B. glycinifermentans* culture. The levels of 2,4,6-THBA were comparatively lower than those of 3,4-DHBA (Figure 4A and Figure S4), while DOPAC was not detected. In contrast, the spent medium from *F. plautii* revealed the presence of DOPAC; however, 2,4,6-THBA, and 3,4-DHBA were undetected (Figure 4B and Figure S4). Consistent with the results obtained in Figure 3, *F. plautii* completely degraded quercetin within 24 h of exposure while *B. glycinifermentans* presented >60% quercetin degradation (Figure 4 and Figure S4).

### 2.3. Degradation Kinetics and Different End Products Suggested Different Mechanisms of Quercetin Degradation by B. glycinifermentans and F. plautii

**Figure 5:**
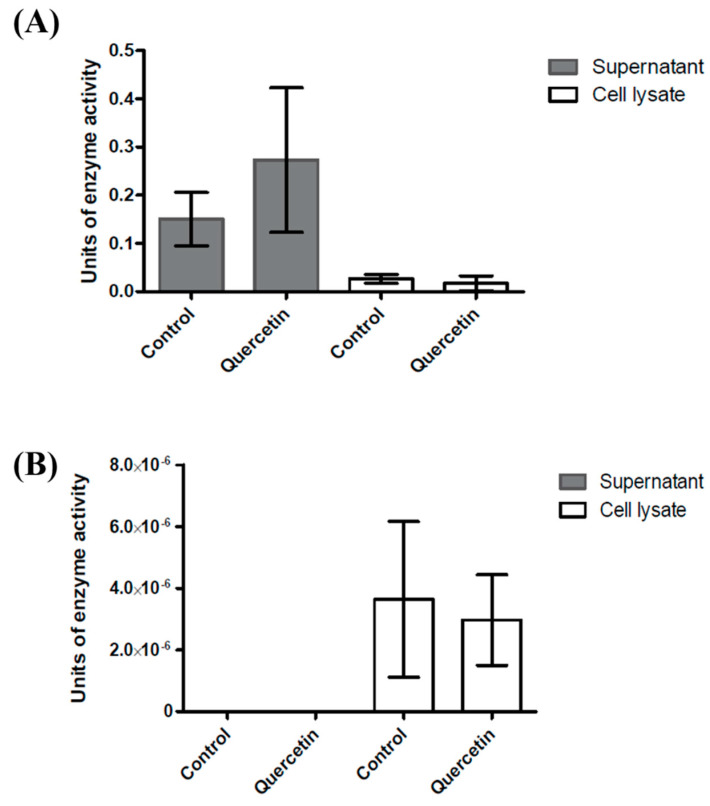
Quercetin-degrading enzyme activity as determined in the culture supernatants and cell lysates of *B. glycinifermentans* (**A**) and *F. plautii* (**B**). As can be observed in the figure, higher enzyme activity was observed in the culture supernatant of *B. glycinifermentans* when compared to the cell lysate (**A**). In contrast, minimal enzyme activity was observed in the cell lysate of *F. plautii,* while there was no quercetin degradation observed in its culture supernatant (**B**). The large standard deviations observed in these experiments are inherent to the nature of the study employed as previously reported [36]; however, the pattern observed is highly reproducible.

The differences in the metabolites produced between *B. glycinifermentans* and *F. plautii* possibly suggest that these bacteria utilize different pathways for quercetin biotransformation. Therefore, we determined the presence of quercetin-degrading enzyme activity in the culture supernatants and the bacterial cell lysates. The bacteria were grown under identical conditions as described in Figure 4, and the supernatants and bacterial cell lysates were obtained. When assayed for quercetin degradation, *B. glycinifermentans* exhibited higher units of enzyme activity in the supernatant when compared to the cell lysate (Figure 5A). In these experiments, one unit of enzyme activity is defined as the amount of enzyme required to convert 1 µmol quercetin in 1 min at 25 °C [36]. Interestingly, the supernatant of *F. plautii* did not exhibit any quercetin-degrading activity; its cell lysate, however, presented minimal enzyme activity (Figure 5B). Taken together, this indicates that *B. glycinifermentans*-mediated degradation of quercetin possibly occurs through a secreted enzyme while *F. plautii* requires a live culture for the biotransformation of quercetin that most likely occurs intracellularly or at the cell surface.

### 2.4. Genetic and Transcriptomic Analysis Revealed the Differential Expression of Genes in B. glycinifermentans and F. plautii upon Exposure to Quercetin

**Figure 6:**
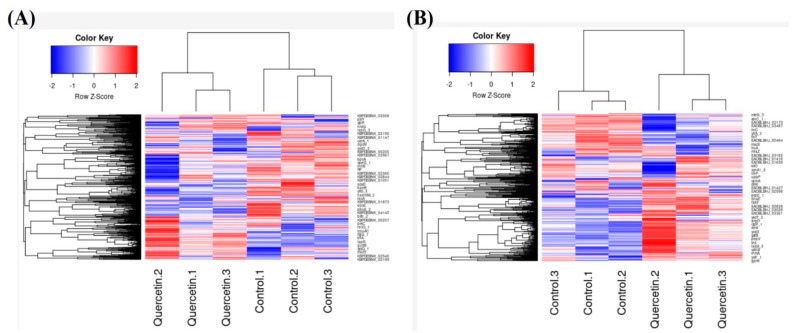
Hierarchical cluster heat map of differentially expressed genes in (**A**) *B. glycinifermentans* and (**B**) *F. plautii*. The figure represents the expression level of each cluster of genes for (**A**) *B. glycinifermentans* and (**B**) *F. plautii* with (QC) and without (X7N) quercetin.

To further understand the molecular basis of quercetin biotransformation, we performed transcriptomic analysis of these two species following quercetin exposure. To this end, the bacteria were grown in the 7NQ media till 50% of the supplemented quercetin was degraded (24 h for *B. glycinifermentans* and 6 h for *F. plautii*). The RNA from the bacterial pellet was then isolated, cDNA was prepared, and the libraries were sequenced using 2 × 300 base chemistry on an Illumina Miseq platform. As can be observed in Figure 6, there was differential gene expression observed in both bacteria in the presence of quercetin as compared to the control. Further analysis indicated that the majority of genes were significantly upregulated in *B. glycinifermentans* while a large majority of genes in *F. plautii* were downregulated upon exposure to quercetin (Figure S5). When twofold differentially expressed genes were mapped on the KEGG metabolic pathways, 74 pathways were found to be modulated in *B. glycinifermentans* following quercetin exposure for 24 h. Among these, 22 pathways had four or more genes mapped to the same pathway (Table 1). Among the differentially expressed pathway genes, sixty genes belonged to the pathways of biosynthesis of secondary metabolites (bgy01100). The majority of these genes (25 genes) were also linked to central carbon metabolism (bgy01200). We detected the expression of pathways for the bacterial secretion system (bgy03070). Within this pathway expression of Ffh (signal recognition particle protein), secA, secDF and secY (protein translocation subunits) were detected. Upregulation of the secretion system apparatus detected here is consistent with our finding of quercetin-degrading activity detected in the cell-free supernatant of *B. glycinifermentans* (Figure 5A) and might be an indication that these secretion system-associated genes may be involved in the secretion of a quercetin-degrading enzyme into the extracellular space.

Our attempts to gain functional insights into the transcriptomic response of *F. plautii* did not yield satisfactory results because the cellular pathways for this species are poorly defined. For example, when the enzymatic function-associated genes from *F. plautii* were searched against the KEGG pathways, only 21 pathways were mapped to the annotated genes list, indicating the need for better genome annotation for this species. When the differentially expressed genes in *F. plautii* were further examined, we found that most genes were downregulated. This is consistent with the fact that *F. plautii* did not grow in the 7NQ medium. Another reason for the low number of differentially expressed genes could be short incubation time in the 7NQ medium (6 h). As the secondary analysis, we determined expression of the known genes associated with quercetin biotransformation. In this regard, three enzymes, quercetin-2,3-dioxygenase (quercetinase), phloretin hydrolase and pirin-like protein, were chosen as they had already been implicated in quercetin degradation [36,37,38]. TBLASTN analysis using known enzyme sequences from other species against *B. glycinifermentans* and *F. plautii* revealed presence of the homologs of all the three proteins in both bacteria (based on >30% sequence identity; Supplemental Table S1).

**Figure 7:**
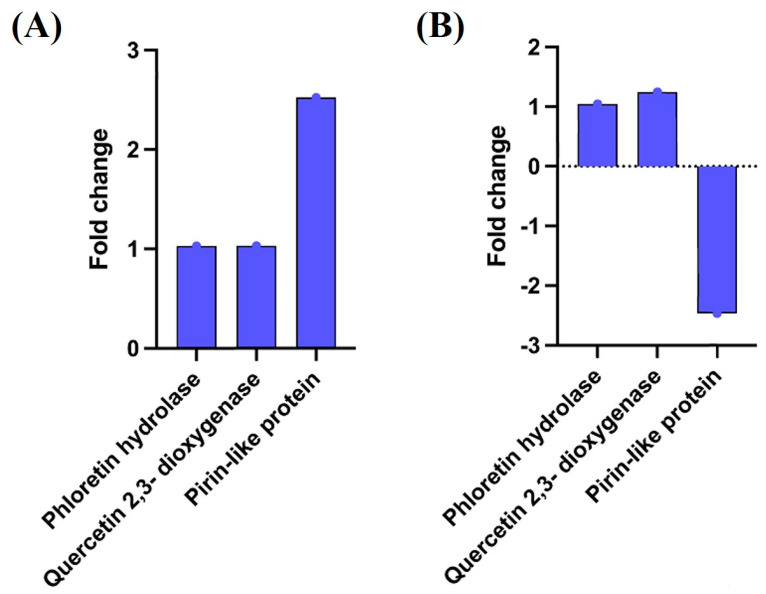
Expression of three different quercetin-degrading enzyme homologs in (**A**) *B. glycinifermentans* and (**B**) *F. plautii* upon exposure to quercetin. The figure shows that the expression of the pirin-like protein was significantly altered in both bacteria in the presence of quercetin. However, no significant changes could be observed in the expression levels of quercetinase and phloretin hydrolase.

Further analysis to understand the expression levels of quercetinase, pirin-like protein and phloretin hydrolase in these samples revealed a more than two-fold expression of pirin-like protein mRNA in *B. glycinifermentans*. In contrast, pirin-like protein mRNA in *F. plautii* was significantly downregulated. The expression levels of quercetinase and phloretin hydrolase in these bacteria were, however, not significantly different from the control (Figure 7).

### 2.5. DOPAC Inhibited Cancer Cell Colony Formation

**Figure 8:**
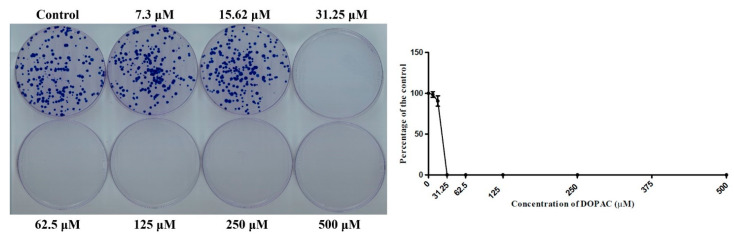
Effect of DOPAC on HCT-116 colony formation. Quantification of the data is shown beside the crystal violet-stained images of the colonies. The graph is represented as the mean ± standard deviation. The concentrations of the drugs used are indicated in μM.

Our group previously demonstrated the ability of 2,4,6-THBA and 3,4-DHBA to inhibit cancer cell colony formation; however, the effect of DOPAC on colony formation was not investigated in that report [24]. Hence, a clonogenic assay was performed to demonstrate the effectiveness of DOPAC against colony formation in the HCT-116 cells. The results indicated that in this cell line, DOPAC inhibited colony formation at the concentrations ≥15.62 μM (Figure 8). The antiproliferative effect of pure DOPAC observed in this study is similar to the observations reported previously in the literature [22].

### 2.6. Clonogenic Assay Using the HCT-116 Cells with the Spent Bacterial Culture Supernatants

**Figure 9:**
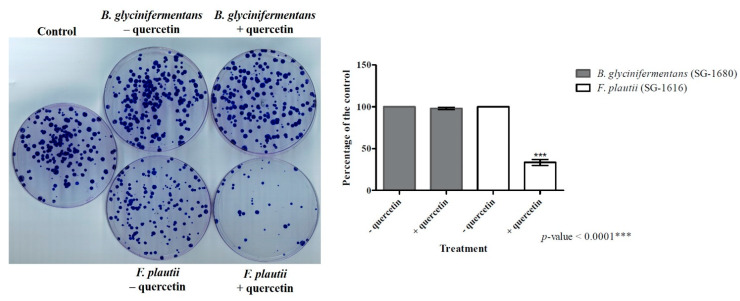
Clonogenic assay using the HCT-116 cells treated with the spent bacterial culture supernatants. Effect of the spent supernatants from the *B. glycinifermentans* and *F. plautii* cultures grown in the absence (− quercetin) and presence (+ quercetin) of quercetin on colony formation in the HCT-116 cells. Quantification of the data is shown beside the crystal violet-stained images of the colonies. The graph is represented as the means ± standard deviation. While in some plates the distribution of the 500 cells seeded was homogeneous, others had clusters of cells localized to certain regions of the plate, owing to their placement in the incubator. All the plates were, however, seeded with 500 cells/plate and allowed to form colonies.

The results obtained in Figure 8 regarding the effect of pure DOPAC on the HCT-116 cells prompted us to examine the growth-inhibitory properties of the spent bacterial culture supernatants on this colon cancer cell line. The bacterial cells were inoculated at the OD_600_ of 0.05 in 25 mL 7N minimal medium with and without quercetin (37.5 μg/mL for *B. glycinifermentans* and 75 μg/mL for *F. plautii*) and grown anaerobically for 24 h. These concentrations were selected to ensure that incubation with the corresponding bacteria for 24 h would result in complete degradation of quercetin (based on the previous data). The culture supernatants were then harvested through centrifugation, and any remaining bacterial cells/particles were removed through filtration (0.22-μm filters). HPLC analysis showed that the supernatants from both bacterial cultures contained only the metabolites and did not contain any quercetin (data not shown), suggesting that any antiproliferative effect observed would most likely be linked to the metabolites. As described in Figure 8, 1 mL sterile culture supernatant was used for the colony formation assay. Following incubation for 14–21 days, with three changes in the medium and treatments, the colonies were stained using crystal violet and counted. As can be observed in Figure 9, the culture supernatant of *F. plautii* grown in the presence of quercetin presented significant inhibition of colony formation (~65% inhibition compared to the supernatant without quercetin), whereas only marginal inhibition was observed in the cells incubated with the culture supernatant of *B. glycinifermentans* containing quercetin (~3% inhibition compared to the supernatant without quercetin).

The minimal effect of the *B. glycinifermentans* supernatant on the HCT-116 cells observed was likely to be due to the lower concentrations of 3,4-DHBA and 2,4,6-THBA as determined through HPLC. Estimation of the metabolites in the culture supernatant showed that 1 mL of the *B. glycinifermentans* supernatant contained 68.93 μg and 16.17 μg of 3,4-DHBA and 2,4,6-THBA, respectively. This roughly translates to 40.66 μM 3,4-DHBA and 8.64 μM 2,4,6-THBA in the volume of 10 mL used for the colony formation assay. It should also be noted here that although 2,4,6-THBA was detected in the culture supernatant, it would not contribute to the inhibition of colony formation in the HCT-116 cells because of the lack of a functional monocarboxylic acid transporter in these cells [24]. Additionally, we also demonstrated in that report that 3,4-DHBA caused inhibition of HCT-116 cell colony formation at the concentration of 250 μM, which was not achieved in this study with 1 mL bacterial culture supernatant (see Figure 9). Hence, methods such as selective enrichment of 3,4-DHBA through chromatography or alternative techniques are required to study its effect on cancer cell colony formation. On the other hand, 1 mL culture supernatant from *F. plautii* contained 20.45 μg DOPAC, which is equivalent to 11.05 μM DOPAC in 10 mL culture medium, and this concentration appears to be sufficient to cause inhibition of cell proliferation.

## 3. Discussion

The flavonoid quercetin has been shown to have health benefits against numerous disorders including inflammation, hypertension, obesity and atherosclerosis [39] and in the prevention of many types of cancers [3,17]. Increasing evidence in the literature suggests that the metabolites of quercetin may be responsible for these observed health benefits. Three observations that support the role of quercetin metabolites in human health include (1) pH-dependent degradation of quercetin in the intestines (in the basic environment), (2) low absorption of quercetin in the intestines resulting in low bioavailability and (3) the biotransforming capability of resident gut microflora. In this regard, the bacteria responsible for the biotransformation of flavonoids such as quercetin are poorly understood. Therefore, we performed screening of the gut microbiota culture collection developed by our group to identify the species responsible for quercetin biotransformation. Our screening yielded five bacteria capable of degrading quercetin. These include *B. glycinifermentans*, *F. plautii*, *B. eggerthii*, *O. scatoligenes* and *E. eligens*. The results presented in this study also demonstrate the ability of select human gut bacteria to generate the metabolites 2,4,6-THBA, 3,4-DHBA and DOPAC. These results now tie in well with our previously published reports where we demonstrated the ability of some of these hydroxybenzoic acid metabolites (2,4,6-THBA, 3,4-DHBA and 3,4,5-THBA) to inhibit cancer cell growth [24]. This report also constitutes the first demonstration of quercetin degradation by *B. glycinifermentans, B. eggerthii, O. scatoligenes* and *E. eligens*. All the five species identified in this study have been demonstrated to be present in human fecal content, suggesting that these species in the gut are capable of biotransforming quercetin. Our demonstration of the ability of *F. plautii* and *Lactobacillus* species (positive control) to degrade quercetin is also consistent with the previously published reports [23,26,35,40].

Although *F. plautii* was previously reported to degrade quercetin to generate DOPAC, the research described in this report for the first time identified the ability of *B. glycinifermentans* to biotransform quercetin to generate 2,4,6-THBA and 3,4-DHBA. *B. glycinifermentans*, which was reported to be part of human fecal content by Ghimire et al. [31], was initially characterized as being present in fermented soybean paste, hence the name *B. glycinifermentans* [33]. Interestingly, based on the analysis of its complete genome, this bacterium was suggested for use as a probiotic for livestock to enhance immune stimulation, enzyme production and pathogen inhibition [34]. The link between our observation that it is capable of biotransforming quercetin to generate 2,4,6-THBA and 3,4-DHBA and its suggested use as a probiotic in the previous report [34] makes this bacterial strain an interesting candidate for the maintenance of human gut health.

The detection of DOPAC as a metabolite of quercetin generated by *F. plautii* in this study confirms the previous reports in literature [35]. In this study, we did not detect the presence of 2,4,6-THBA and 3,4-DHBA in the spent medium from *F. plautii*. Although the reason for this is currently unclear, it is possible that the amounts of the other metabolites (2,4,6-THBA and 3,4-DHBA) generated from this bacterium might have been below the detection levels of the HPLC technique employed. Similarly, the lack of detection of DOPAC in the spent medium from *B. glycinifermentans* may be attributed to its low levels in the samples. It is to be noted that TBLASTN analysis revealed the presence of homologs of quercetinase, phloretin hydrolase and pirin-like protein in both *B. glycinifermentasns* and *F. plautii*. Consistent with this, both species exhibited the presence of transcripts for quercetinase and phloretin hydrolase; however, the abundance of pirin-like protein implicated in quercetin degradation appears to be differentially regulated with a more than two-fold increase in *B. glycinifermentans* and a more than two-fold decrease in *F. plautii* (Figure 7). This differential regulation may account for the differences in the metabolites produced as detected by HPLC. Alternatively, it is also possible that the quercetin degradation pathway utilized by *F. plautii* is radically different from that of *B. glycinifermentans* and hence generates different metabolites. This is supported by the observation that the spent culture supernatant from *B. glycinifermentans* had quercetin-degrading enzyme activity while the culture supernatant of *F. plautii* did not (Figure 5). This observation also suggests that the quercetin-degrading enzymes in *B. glycinifermentans* and *F. plautii* are likely to be differentially localized (secreted vs. membrane-bound/intracellular). Furthermore, the degradation kinetics demonstrates that *F. plautii* completely degrades quercetin within 12 h of incubation, whereas *B. glycinifermentans* requires around 48 h for complete quercetin degradation. This, along with the minimal enzyme activity detected in the cell lysate (Figure 5B), suggests that the degradation of quercetin by *F. plautii* may require the presence of live bacterial cells. A recent study by Yang et al. demonstrated the involvement of ene-reductase, chalcone isomerase, enoate reductase and phloretin hydrolase in the generation of metabolites from flavones and flavonols [28]. Hence, the lack of quercetin degradation in the culture supernatant and minimal activity in the pellet observed in our study (Figure 5B) may have also been related to the requirement of four different enzymes for the generation of DOPAC from quercetin. Alternatively, it is also possible that the enzymes involved in *F. plautii* are sensitive to the experimental conditions or require other cofactors when assayed in vitro, which may not be the case for *B. glycinifermentans*. The observation that pirin-like protein, which was previously reported to have quercetin-2,3-dioxygenase activity [37], was differentially expressed in *B. glycinifermentans* and *F. plautii* may provide a link to the differences observed in the generation of metabolites between the two species of bacteria. Hence, further research should shed light on the specific pathways utilized by these bacteria for quercetin biotransformation.

The importance of 2,4,6-THBA and 3,4-DHBA in the inhibition of cancer cell growth was well-established by our group previously [24], and in the present study, we demonstrated that *F. plautii* is able to produce DOPAC at concentrations sufficient to produce an antiproliferative effect on cancer cells when tested ex vivo. It is to be noted that DOPAC has also been shown to inhibit cancer cell proliferation by other investigators in various cancer cell types [22,26,41]; this is believed to occur through its antioxidant properties. The demonstration of the ability of the bacterial culture supernatant of *F. plautii* grown in the presence of quercetin to inhibit colony formation is, to our knowledge, the first report showing the direct effect of bacteria-generated metabolites on cancer cell growth and, therefore, is a very significant finding. We observed that while 1 mL supernatant from the *F. plautii* culture was sufficient to inhibit colony formation in the HCT-116 cells, 1 mL culture supernatant from the *B. glycinifermentans* was insufficient to exert a similar inhibitory effect. Quantification of the metabolites indicated that the amount of 3,4-DHBA generated in our experiments from *B. glycinifermentans* was lower (40.66 μM) than that required for effective inhibition (250 μM) [24]. As the addition of >1 mL bacterial culture supernatant to 10 mL cell culture medium may affect osmolarity of the medium and, in turn, cancer cell growth in the culture, the effect of larger volumes of bacterial supernatants on colony formation was not tested. As an alternative strategy, we performed concentration of the supernatants through rota-evaporation at 65 °C under vacuum conditions. Addition of the 5× and 10× concentrated samples dissolved in the cell culture medium failed to inhibit cancer cell growth (data not shown) for the supernatant obtained from the *F. plautii* culture. We believe that this could be related to the instability of DOPAC during the process of concentration; for example, temperature may affect the stability of DOPAC. Therefore, alternative methods of concentration need to be explored to demonstrate the effectiveness of bacterial supernatants where metabolites are generated at lower concentrations (such as for *B. glycinifermentans*) against cancer cell growth.

While the human gut is known to harbor 300–500 species of bacteria [42,43], this study investigated the potential of only 94 bacterial species to degrade quercetin. Therefore, additional screening is required to establish the contribution of other bacterial strains to CRC prevention as well. However, it is interesting to note that of the 94 strains screened, only five exhibited the ability to degrade quercetin. This suggests that the flavonoid-biotransforming ability may be narrowly restricted to only a few species of bacteria, highlighting the importance of these bacteria in the prevention of CRC. While the focus of our study was on quercetin, it is to be noted that the diet also contains other flavonoid members (such as anthocyanins, epigallocatechin gallate, catechins, cyanidin-3-glucoside, etc.) that may be biotransformed by the gut microflora [44]. For example, DOPAC, 2,4,6-THBA and 3,4-DHBA have been reported to be metabolites produced upon green and black tea consumption [22,45] while 2,4,6-THBA and 3,4-DHBA have been demonstrated to be generated upon the consumption of the anthocyanin, cyanidin-3-glucoside [9]. Additionally, phenolic acids have also been shown to be produced from the intestinal degradation of fibers by colonic bacteria [46].

In this study, the bacterial strains were grown individually to screen for their ability to degrade quercetin, but it is still unknown how quercetin may be degraded in the presence of other bacteria, in cocultures. It is known that some bacterial species influence the growth of others; diet is also suggested to contribute to this selection [44,47]. Therefore, additional studies are required to establish how diet influences the growth of these species and overall degradation of quercetin in vivo. It will be interesting to explore the metabolism of quercetin by other species of bacteria, enzymes involved in this process and characterize the metabolites generated individually or in a community setting. Additional studies are also required to test the bacterial culture supernatants containing these metabolites for their ability to inhibit cancer cell proliferation.

## 4. Materials and Methods

### 4.1. Bacterial Culture Medium

Modified Brain Heart Infusion (mBHI) broth was prepared as described by Ghimire et al. [31], with minor modifications, and this medium was used as the base medium for growing the strains. Briefly, yeast extract (5.0 g/L), L-cysteine (0.3 g/L), 1 mL/L resazurin (0.25 mg/mL) and 1 mL/L menadione (5.8 mM) were added to the standard BHI base ingredients. The prepared medium was purged with nitrogen gas until it became completely anaerobic. The medium was then autoclaved at 121 °C and 15 psi for 30 min. The sterilized medium was then supplemented with 1 mL/L hemin solution (0.5 mg/mL), 10 mL/L ATCC vitamin mixture (ATCC, Manassas, VA, USA) and 10 mL/L ATCC mineral mixture (ATCC, Manassas, VA, USA).

For the experiments studying quercetin degradation, 7N minimal medium was used as described by Rodrigus-Castano et al. [48], with slight modifications. Briefly, 50 mM MOPS⋅NaOH (pH 7.2), 0.02% resazurin, 2 mM tricine, 0.025% Tween 80, 20 mM CH_3_COONa, 20 mM NaCl, 14 mM NH_4_Cl, 0.25 mM K_2_SO_4_, 0.5 mM MgCl_2_⋅6H_2_O, 0.5 mM CaCl_2_⋅2H_2_O, 10 μM FeSO_4_⋅7H_2_O, 20 mM NaHCO_3_, 1 mM KH_2_PO_4_, 1 μg/mL menadione, 1.9 μM hemin, 0.2 mM histidine, 8 mM L-cysteine and 40 mM D-glucose were added, and the prepared medium was purged with nitrogen until it became completely anaerobic. Following this, the medium was supplemented with 1× ATCC trace minerals and 1× ATCC vitamin supplements. The medium was then filter-sterilized by filtration using a 0.22-μm nylon membrane filter. Quercetin was dissolved in ethanol (75 mg/mL) and added to this medium to obtain the final concentration of 75 µg/mL to give a 7NQ medium.

### 4.2. Screening for Bacterial Species Capable of Metabolizing Quercetin

We first analyzed 91 human gut species from the human gut bacterial library [31] for their ability to grow in a 7NQ medium. Briefly, the liquid culture of each strain was adjusted to the OD_600_ of 0.5, and 20 µL of this culture were inoculated into 1 mL of a 7NQ medium. The bacterial culture was then grown for 48 h anaerobically. Following incubation, the OD_600_ of the strains was measured and compared to their growth in a 7 N medium. We used HPLC to analyze the ability of the tested bacteria to degrade quercetin.

### 4.3. DPH Assay

Stock solutions of 1,6-diphenyl-1,3,5-hexatriene (DPH) and quercetin were prepared in DMSO at the concentrations of 1 mM and 20 mM, respectively. The bacterial strains that presented the quercetin-degrading ability from the HPLC analysis and *E. coli* (as the negative control) were grown anaerobically at 37 °C in an mBHI medium for 24–48 h. These strains were then streaked on mBHI agar plates containing a circular nylon membrane soaked in a mixture containing equal volumes of 1 mM DPH and 20 mM quercetin. The plates were then incubated anaerobically at 37 °C for 24–48 h and visualized under UV light using BioRad’s (Hercules, CA, USA) ChemiDocXRS+ gel documentation system.

### 4.4. UV-Visible Absorbance Spectroscopy

UV-visible absorbance spectroscopy was used to analyze the quercetin biotransformation products in the spent medium. UV-visible spectra were recorded from 600 nm to 200 nm with a Varian Cary 3 E UV-visible spectrophotometer using samples in 1-cm path length quartz cuvettes at 25 °C. Spectra of the standards were taken in DMSO, of the metabolites resulting from the biotransformation of quercetin—in the 7N medium. The spectra were normalized and plotted using the OriginPro graphing and analysis software.

### 4.5. HPLC Analysis for the Detection of Quercetin/Metabolites in Bacterial Culture Supernatants

High-Performance Liquid Chromatography (HPLC) was utilized to determine the concentration of quercetin in the samples. Ethyl acetate and the 7NQ medium were taken in a ratio of 2:1, and the samples were vortexed thoroughly, following which phase separation was achieved by centrifuging the samples at 14,000× *g* rpm for 5 min. The organic phase was aspirated to a fresh tube and dried under a steady stream of argon gas. Following this, 400 μL of the mobile phase were added to the dried samples and vortexed to mix the contents. The samples were centrifuged at 14,000× *g* rpm for 5 min to pellet any debris, and the supernatants were used for HPLC analysis. HPLC analysis was conducted using an Agilent HPLC system equipped with a PDA detector. An isocratic method was used to elute the compound in the reversed phase using an Agilent (Santa Clara, CA, USA) ZORBAX (5 µm, 4.6 × 250 mm) column. The mobile phase for quercetin detection consisted of acetonitrile and 2% acetic acid (40:60 ratio), with a flow rate of 0.8 mL/min. The injection volume was 40 µL. Quercetin was detected at the wavelength of 370 nm. Quantification was performed by generating a standard graph using known amounts of quercetin.

The metabolites of quercetin degradation were extracted with double the volume of n-butanol from the spent media. The n-butanol extract was dried under a steady stream of argon gas and the dried samples were dissolved in the mobile phase. The mobile phase for metabolite detection consisted of acetonitrile and 30 mM ammonium acetate (pH 4.0; 89:11 (*v*/*v*)). This solution was added to methanol at the 80:20 ratio. The HPLC apparatus and column used were the same as those used for quercetin detection. The flow rate for the method was maintained at 0.8 mL/min. The injection volume was 40 µL. DOPAC, 2,4,6-THBA and 3,4-DHBA were detected at the wavelength of 270 nm. Quantification was performed by generating a standard graph using known amounts of each metabolite.

### 4.6. Lysis of Bacteria and Assay for Determination of Quercetin Degrading Enzyme Activity

Bacterial cell lysates were prepared as described previously, with minor modifications [36]. Briefly, the cells were inoculated in the 7N (control) and 7NQ media to the OD_600_ of 0.05 and then cultivated anaerobically at 37 °C for 24 h. The cells were then harvested, washed twice with buffer A (50 mM Tris-Cl, pH 7.5, 20 mM NH_4_Cl, 1% *v*/*v* glycerol and 100 mM NaCl) and then lysed by suspension in buffer A containing 0.5 mg/mL lysozyme and 2 units/mL DNase I followed by incubation at 37 °C for 20 min with occasional vortexing. The cell suspension was then centrifuged at 17,000× *g* for 30 min and the supernatants were recovered as crude lysates.

Quercetin-degrading enzyme activity was analyzed by measuring the decrease in the absorbance of the reaction mixture at 367 nm as previously described [36]. Each reaction was set up as follows: 30 µL 45 mM quercetin dissolved in DMSO was added to 870 µL 50 mM Tris-Cl buffer (pH 7.5) and preincubated for 5 min. The enzymatic reaction was started by adding 100 µL of either the spent culture supernatant or the cell lysate and the absorbance was recorded at 367 nm following 2-min incubation at 25 °C. The molar extinction coefficient was calculated using blank samples under the same conditions as the reaction mixture.

### 4.7. Bacterial Whole Genome Sequencing

For genomic DNA isolation, *B. glycinifermentans* and *F. plautii* were grown overnight anaerobically in the mBHI media and genomic DNA was extracted from the pellet using a DNeasy Powersoil Pro kit (Qiagen, Hilden, Germany) according to the manufacturer’s protocol.

The sequencing libraries were prepared with a Nextera XT kit and sequenced with Illumina 2 × 300 paired-end sequencing chemistry on a MiSeq platform. The raw reads and sequencing adaptors were filtered for quality using PRINSEQ, following which they were assembled de novo with a Unicycler [49]. Gene calling was performed utilizing Prokka [50] using the minimum ORF length of 100 bp.

### 4.8. RNA Isolation and Transcriptomic Analysis

The effect of quercetin on bacterial gene expression and regulation was determined through whole transcriptome analysis. Total RNA was extracted from the bacteria grown in the presence or absence (control) of quercetin supplemented at the concentration of 75 µg/mL. *B. glycinifermentans* and *F. plautii* samples were harvested after 24 h and 6 h post-inoculation, respectively, to reflect the time required to degrade 50% of the supplemented quercetin. After growth, 10 mL of these cultures were added to a 50 mL falcon tube, treated with an equal amount of the Bacterial RNA Protect reagent (Qiagen) and incubated at 25 °C for 10 min. The samples were then centrifuged at 5000× *g* for 10 min at 4 °C. The supernatants were discarded, and the total RNA was extracted from the pellet using an RNeasy Plus Mini kit (Qiagen, Hilden, Germany) as per the manufacturer’s instructions, with slight modifications. Briefly, the pelleted cells were dissolved in 200 μL lysis buffer (30 mM Tris, 10 mM EDTA, 20 mg/mL lysozyme, 10 μL Proteinase K (Qiagen, Hilden, Germany), 40 U Mitomycin) and incubated at 25 °C for 40 min. Buffer RLT in the amount of 700 μL was added to this mixture, and the samples were bead-beaten in a 2-mL safe-lock tube containing ~50 mg of zirconia beads (diameter, 0.1 mm; BioSpec Products, Bartlesville, OK, USA) at the maximum speed for 10 min. The lysate was further processed as per the manufacturer’s instructions for RNA isolation.

Large ribosomal RNA was then removed from the total RNA using a RiboMinus Transcriptome Isolation Kit (Invitrogen, Carlsbad, CA, USA) as per the manufacturer’s instructions. Any DNA impurities in the remaining RNA samples were then eliminated by incubating the samples with a DNase I reaction mixture composed of 5 μL DNAse I (Qiagen), 155 μL nuclease-free water and 20 μL DNA digestion buffer at 25 °C for 15 min. Then, mRNA was concentrated using an RNeasy MinElute Cleanup kit (Qiagen, Hilden, Germany) as per the manufacturer’s instructions. Concentration of the RNA samples was then estimated using Qubit 4. The samples were then stored at −80 °C until further use.

### 4.9. Synthesis and Sequencing of cDNA

Using random primers (NEB S1330S), 75 ng rRNA-depleted total RNA (see above) was reverse-transcribed according to the manufacturer’s protocol (Protoscript II, NEB). The synthesized first stand was utilized for the Ultra II Directional RNA library construction (NEBNext^®^ Ultra™). The sequencing library was prepared from 0.3 ng enriched DNA with a Nextera XT library preparation kit (Illumina, San Diego, CA, USA) and sequenced using 2 × 300 base chemistry following the manufacturer’s protocol. Finally, the reads were trimmed, quality-checked and analyzed using a CLC Genomics workbench [51]. A previously sequenced and Prokka-annotated custom genome was used for mapping genes from RNAseq. The Reads Per Kilobase of transcript per Million mapped reads (RPKM) values obtained were exported, and RStudio [52] was used for analysis and graphical representation of the results.

### 4.10. Clonogenic Assay

Clonogenic assays were performed as previously described [53]. Cancer cell lines were seeded at the density of 500 cells/100-mm plate and grown for 48 h, following which the specified compounds were added at the concentrations indicated. Alternatively, where indicated, the HCT-116 cells were treated with 1 mL sterile bacterial culture supernatant. The spent medium was replaced with a fresh medium containing the respective compounds or the bacterial culture supernatant every 5–6 days. The cells were incubated for 14–21 days, fixed with 100% methanol for 20 min and stained with 0.5% crystal violet prepared in 25% methanol. The colonies were then photographed and quantified using NIH ImageJ.

### 4.11. Statistical Analysis

All the experiments were repeated 3–6 times independently of each other. The dataset followed normal distribution with homogeneity of variance, therefore, one-way ANOVA followed by Tukey’s post-hoc tests were adopted to compare group differences to the control, and significance was defined as *p* < 0.05.

## 5. Conclusions

The research described in this report identified five species of bacteria capable of degrading quercetin to give different bioactive metabolites, some of which have been previously characterized to have antiproliferative effects against cancer cells. This study also established clear differences between two bacterial species (*B. glycinifermentans* and *F. plautii*) in terms of their ability to degrade quercetin; in addition, it also showed the generation of different metabolites. We also demonstrated for the first time the inhibitory effect of the bacterial culture supernatant from *F. plautii* against cancer cell growth, paving the way for similar studies with other bacterial culture supernatants. We believe that bacteria-mediated biotransformation of flavonoids and generation of bioactive metabolites are important contributors to colorectal cancer prevention observed in flavonoid-rich diets.

## Figures and Tables

**Table 1:** Differentially expressed pathways in *B. glycinifermentans* following exposure to quercetin. A pathway was considered differentially expressed if four or more ≥ two-fold genes were mapped to the pathway.

Pathway ID	Pathway Name	Number of Differentially Expressed Genes
bgy01110	Biosynthesis of secondary metabolites	60
bgy03010	Ribosome	45
bgy01120	Microbial metabolism in diverse environments	30
bgy01200	Carbon metabolism	25
bgy01230	Biosynthesis of amino acids	23
bgy00230	Purine metabolism	20
bgy01240	Biosynthesis of cofactors	20
bgy00190	Oxidative phosphorylation	13
bgy00010	Glycolysis/gluconeogenesis	12
bgy00250	Alanine, aspartate and glutamate metabolism	9
bgy03018	RNA degradation	9
bgy00680	Methane metabolism	8
bgy00680	Citrate cycle (TCA cycle)	8
bgy00030	Pentose phosphate pathway	7
bgy02024	Quorum sensing	7
bgy00630	Glyoxylate and dicarboxylate metabolism	6
bgy00620	Pyruvate metabolism	6
bgy00260	Glycine, serine and threonine metabolism	5
bgy01210	2-oxocarboxylic acid metabolism	5
bgy00670	One carbon pool by folate	5
bgy02020	Two-component system	4
bgy02010	ABC transporters	4

## Data Availability

The raw RNA-seq data for *B. glycinifermentans* and *F. plautii* were deposited in the NCBI sequence read archive under bioprojects PRJNA733198 and PRJNA733200, respectively.

## Supplementary Materials

The following are available online at https://www.mdpi.com/article/10.3390/ijms22137045/s1, Figure S1: HPLC chromatograms to detect quercetin degradation for the five bacterial species obtained from the human gut library, Figure S2: Growth curves for *B. glycinifermentans* (A) and *F. plautii* (B) in the 7NQ media, Figure S3: UV-visible spectra of the standards and spent media to identify the metabolites resulting from biotransformation of quercetin by *B. glycinifermentans* and *F. plautii*. Metabolites from *B. glycinifermentans* in the 7N medium (black), metabolites from *F. plautii* in the 7 N medium (pink), quercetin in DMSO (red), 3,4-DHBA in DMSO (blue), 2,4,6-THBA in DMSO (cyan), DOPAC in DMSO (green), Figure S4: HPLC chromatograms to detect quercetin degradation by *B. glycinifermentans* and *F. plautii*, Figure S5: Volcano plot showing the differential transcriptome response to quercetin by *B. glycinifermentans* (A) and *F. plautii* (B), Table S1: TBLAST search results to identify homologs of known quercetin-biotransforming genes in *F. plautii* and *Bacillus glycinifermentans* genomes, Table S2: Transcriptomic analysis of *B. glycinifermentans* after exposure to quercetin for 24 h, Table S3: Transcriptomic analysis of *F. plautii* after exposure to quercetin after six hours.
