# Supplementary figures and images for: Screening of Human Gut Bacterial Culture Collection Identifies Species That Biotransform Quercetin into Metabolites with Anticancer Properties
Source: Int J Mol Sci. 2021 Jun 30;22(13):7045. doi: 10.3390/ijms22137045 (PMC8269047; doi:10.3390/ijms22137045)

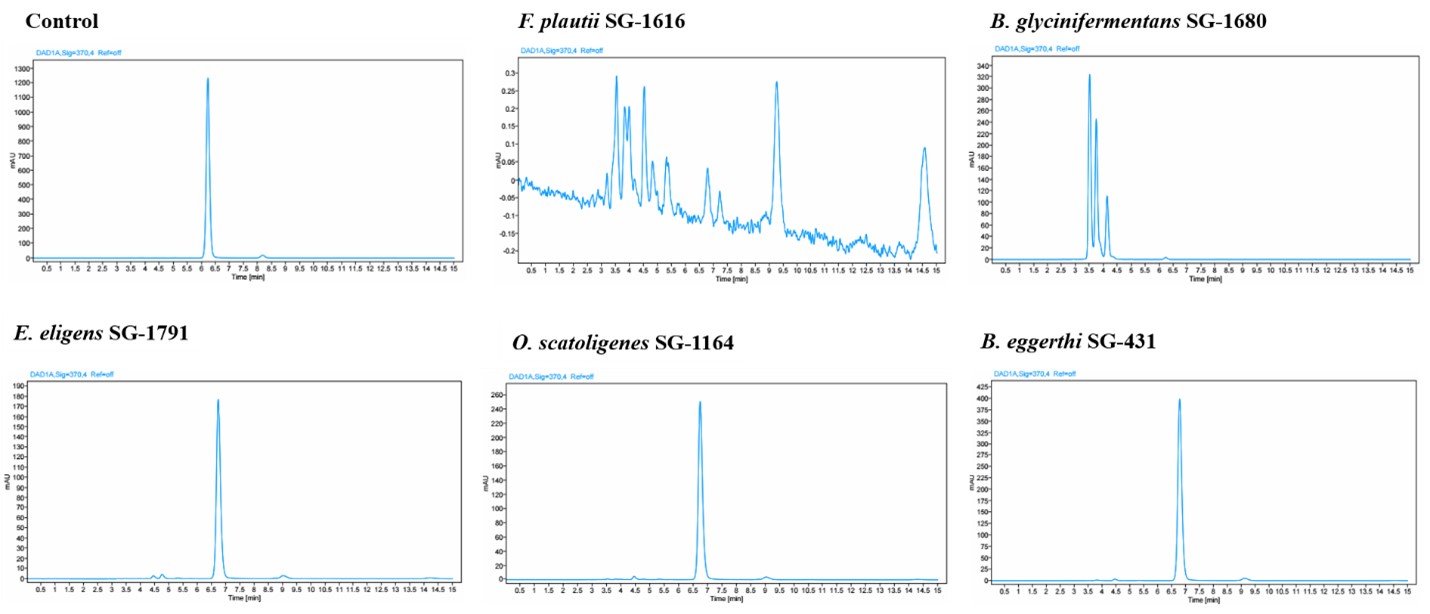

Supplement: Supplementary file 1 [file ijms-22-07045-s001.zip › Figure S1.jpg]

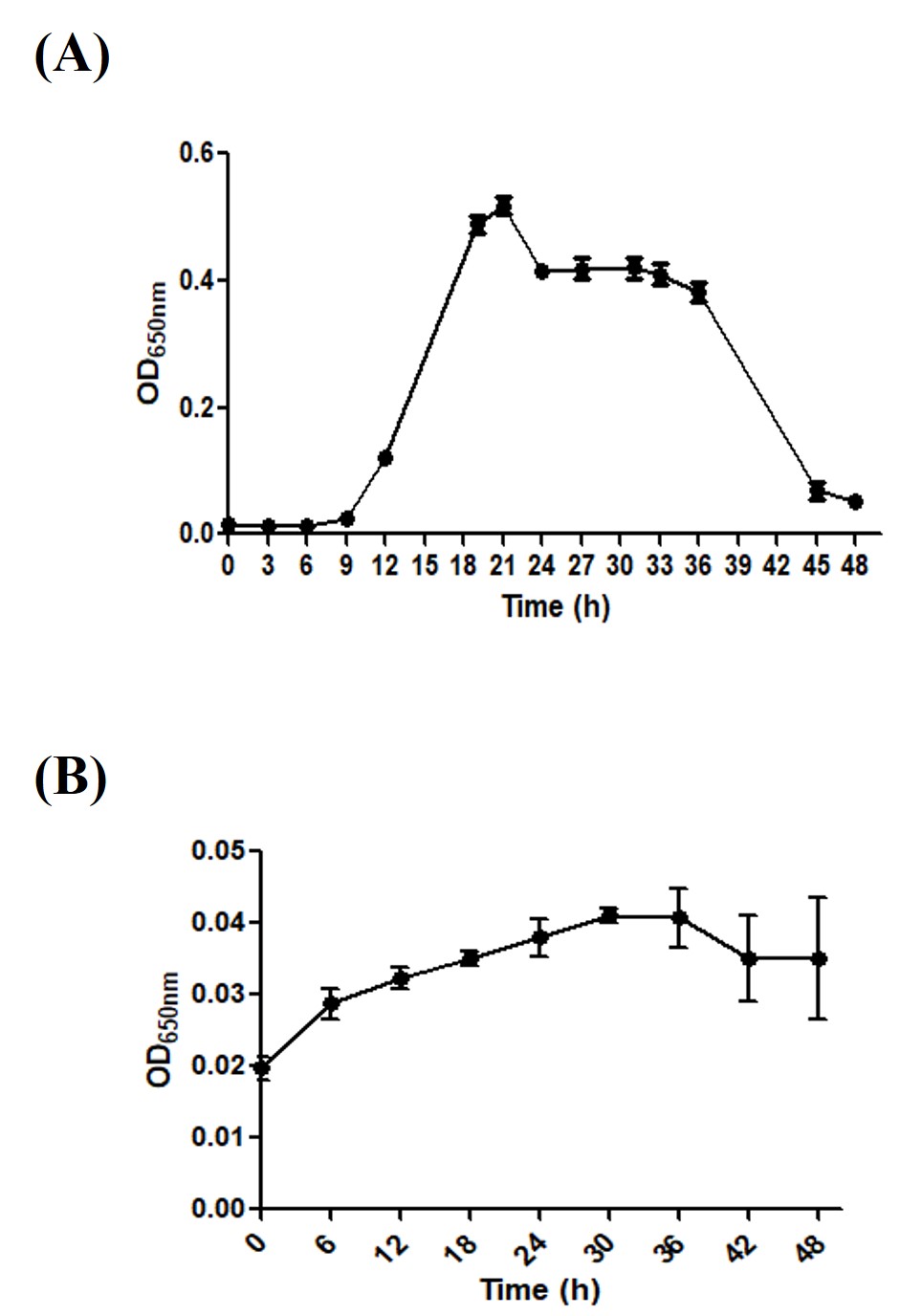

Supplement: Supplementary file 1 [file ijms-22-07045-s001.zip › Figure S2.jpg]

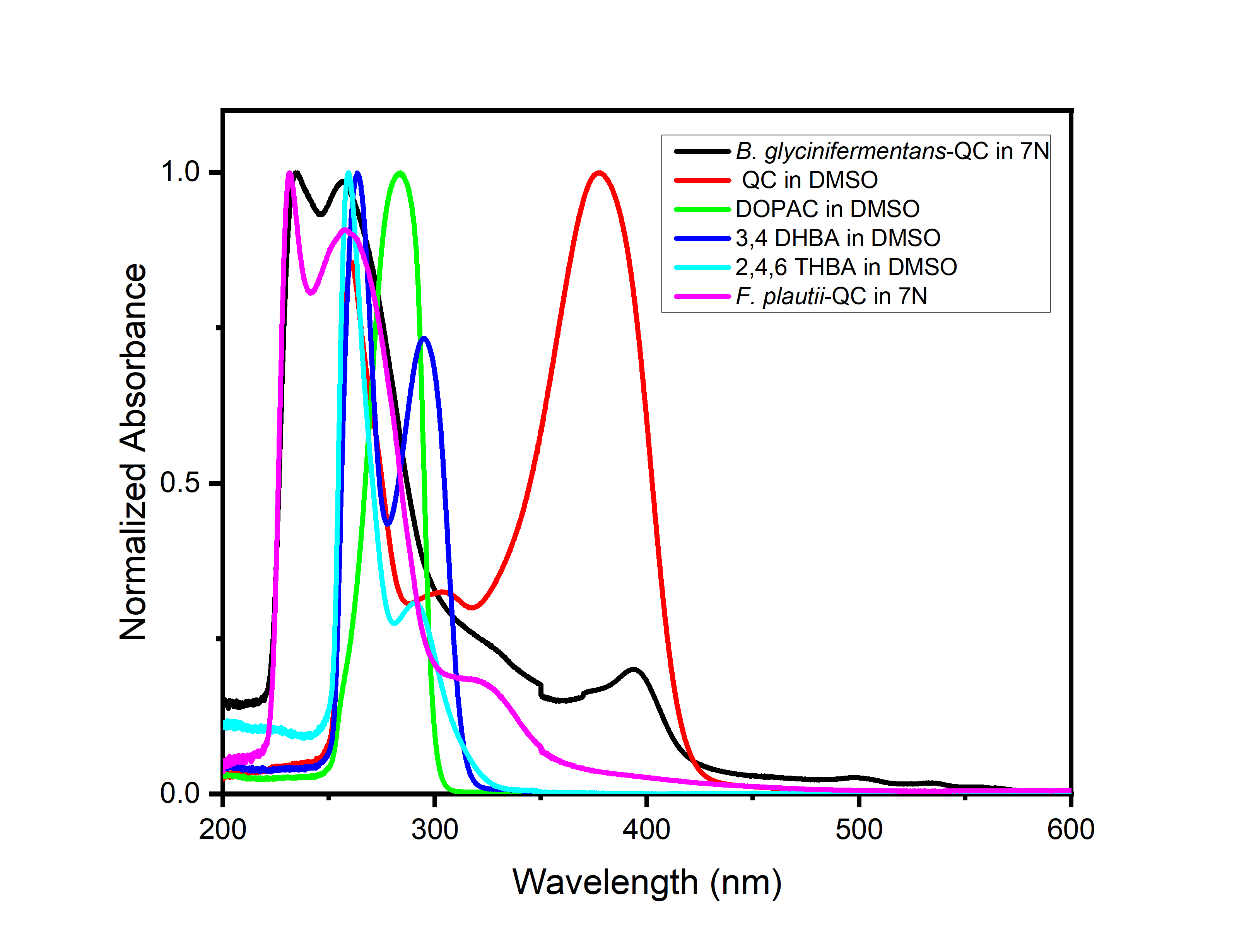

Supplement: Supplementary file 1 [file ijms-22-07045-s001.zip › Figure S3.jpg]

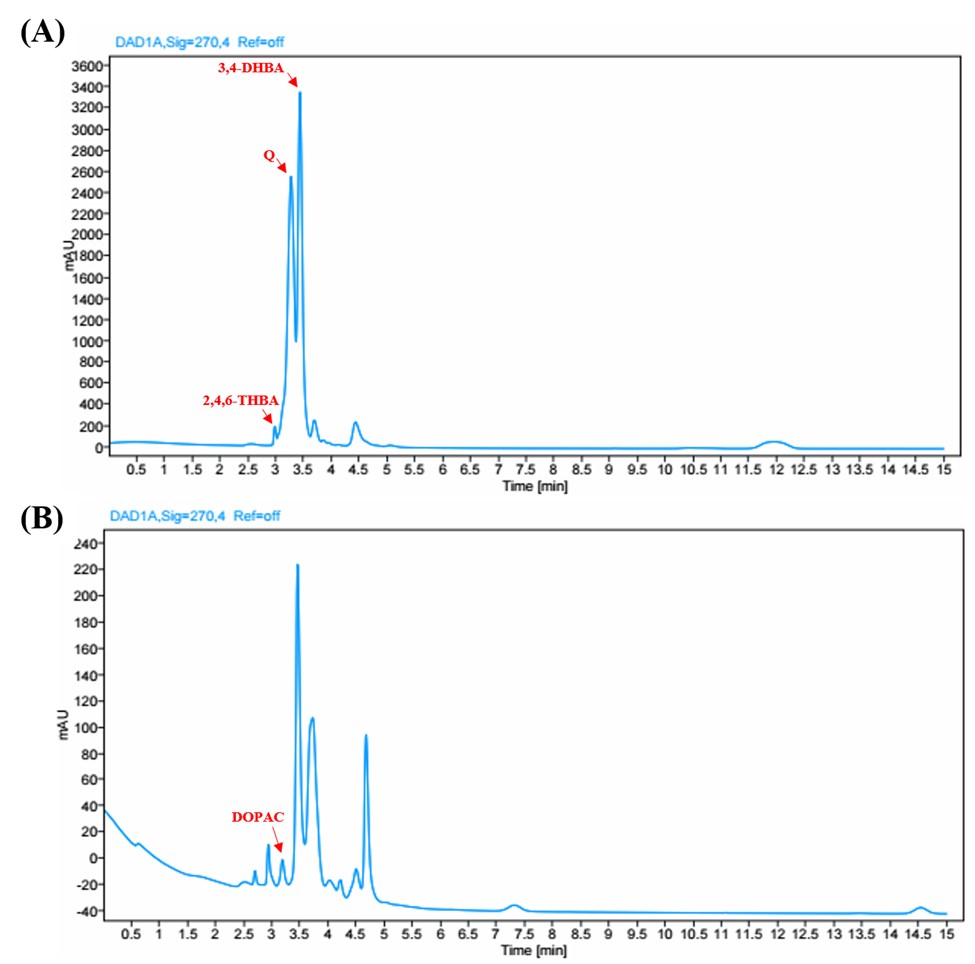

Supplement: Supplementary file 1 [file ijms-22-07045-s001.zip › Figure S4.jpg]

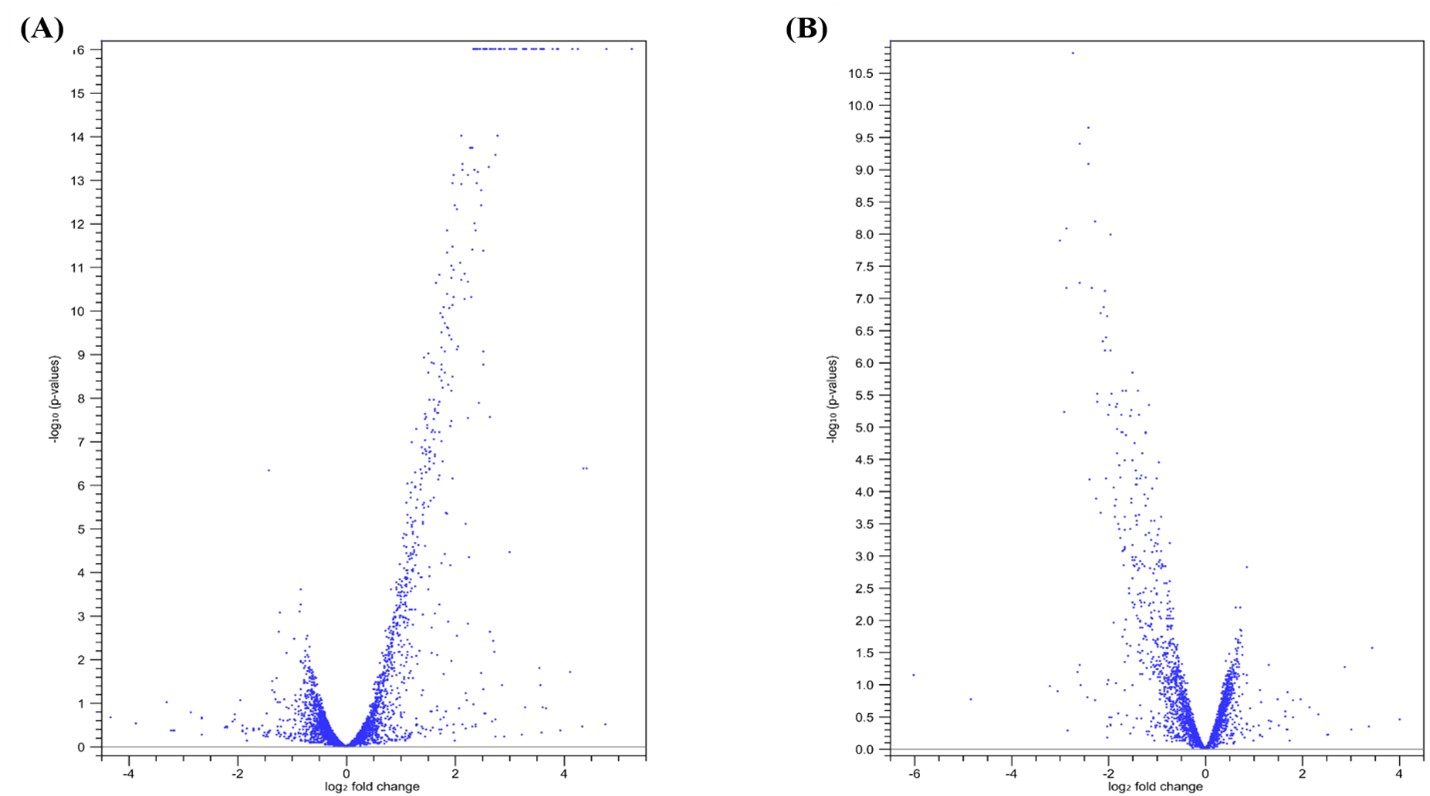

Supplement: Supplementary file 1 [file ijms-22-07045-s001.zip › Figure S5.jpg]
